# Retraction and replacement of: Altered microstructure of the contralesional ventral premotor cortex and motor output after stroke

**DOI:** 10.1093/braincomms/fcae149

**Published:** 2024-05-28

Paweł P Wróbel, Stephanie Guder, Jan F Feldheim, José A Graterol Pérez, Benedikt M Frey, Chi-un Choe, Marlene Bönstrup, Bastian Cheng, Yogesh Rathi, Ofer Pasternak, Götz Thomalla, Christian Gerloff, Martha E Shenton, Robert Schulz, Altered microstructure of the contralesional ventral premotor cortex and motor output after stroke, *Brain Communications*, Volume 5, Issue 3, 2023, fcad160, https://doi.org/10.1093/braincomms/fcad160

Subsequent to the publication of the above article, secondary analyses by the authors themselves led to the detection of a major error during preprocessing and data analysis, which the authors reported to the Journal. As a result, this article is being retracted. The authors have corrected their honest error and all analyses have been repeated. A new version of this article was submitted, reviewed and accepted by the editor in chief, and the new version has been republished as https://doi.org/10.1093/braincomms/fcae115. Note that the previously positive findings were no longer detected.

